# Nutritional Interventions to Attenuate Quadriceps Muscle Deficits following Anterior Cruciate Ligament Injury and Reconstruction

**DOI:** 10.1007/s40279-025-02174-w

**Published:** 2025-01-24

**Authors:** Miriam J. Smith, Nolan J. Hoffman, Argell J. San Jose, Louise M. Burke, David A. Opar

**Affiliations:** 1 School of Behavioural and Health Sciences, Australian Catholic University, Melbourne, VIC Australia; 2 Sports Performance, Recovery, Injury and New Technologies (SPRINT) Research Centre, Australian Catholic University, Melbourne, VIC Australia; 3 Exercise and Nutrition Research Program, Mary MacKillop Institute for Health Research, Australian Catholic University, Melbourne, VIC Australia; 4 OrthoSport Victoria Institute (OSVi), Richmond, VIC Australia; 5 Level 1, Daniel Mannix Building, 17 Young Street, Fitzroy, VIC 3065 Australia

## Abstract

Following anterior cruciate ligament (ACL) injury, quadriceps muscle atrophy persists despite rehabilitation, leading to loss of lower limb strength, osteoarthritis, poor knee joint health and reduced quality of life. However, the molecular mechanisms responsible for these deficits in hypertrophic adaptations within the quadriceps muscle following ACL injury and reconstruction are poorly understood. While resistance exercise training stimulates skeletal muscle hypertrophy, attenuation of these hypertrophic pathways can hinder rehabilitation following ACL injury and reconstruction, and ultimately lead to skeletal muscle atrophy that persists beyond ACL reconstruction, similar to disuse atrophy. Numerous studies have documented beneficial roles of nutritional support, including nutritional supplementation, in maintaining and/or increasing muscle mass. There are three main mechanisms by which nutritional supplementation may attenuate muscle atrophy and promote hypertrophy: (1) by directly affecting muscle protein synthetic machinery; (2) indirectly increasing an individual’s ability to work harder; and/or (3) directly affecting satellite cell proliferation and differentiation. We propose that nutritional support may enhance rehabilitative responses to exercise training and positively impact molecular machinery underlying muscle hypertrophy. As one of the fastest growing knee injuries worldwide, a better understanding of the potential mechanisms involved in quadriceps muscle deficits following ACL injury and reconstruction, and potential benefits of nutritional support, are required to help restore quadriceps muscle mass and/or strength. This review discusses our current understanding of the molecular mechanisms involved in muscle hypertrophy and disuse atrophy, and how nutritional supplements may leverage these pathways to maximise recovery from ACL injury and reconstruction.

## Key Points

**Table:** 

Resistance training is known to stimulate key signalling pathways within skeletal muscle involved in muscle hypertrophy. However, quadriceps muscle atrophy persists following anterior cruciate ligament (ACL) injury despite rehabilitative resistance training.
Nutritional supplementation may support muscle hypertrophy following ACL injury and reconstruction in three ways: (1) by exerting a direct effect on muscle protein synthesis; (2) indirectly, by increasing the individual’s ability to work harder and complete rehabilitation training more effectively; and (3) through a direct effect on satellite cell proliferation and differentiation. This review highlights potential roles for nutritional supplementation in promoting quadriceps muscle hypertrophy following ACL injury and reconstruction, when used together with resistance training.

## Introduction

Over the last 20 years there has been a notable global increase in the incidence of injuries to the anterior cruciate ligament (ACL) [1–3], with the long-term economic burden estimated to be as high as USD $7.6 billion per year in the USA alone [4]. One of the major consequences of ACL injury and reconstruction is the accelerated deterioration of knee joint cartilage, resulting in osteoarthritis in more than 50% of cases [5], increasing the likelihood of complete knee replacement [6].

While the direct trauma of an ACL injury is sustained by ligamentous tissue, atrophy of the quadriceps muscles following ACL injury and reconstruction is well documented, and can be associated with persistent deficits in quadriceps strength and function even after rehabilitation [7, 8]. Deficits in quadriceps muscle mass have been associated with a number of long-term consequences in individuals with prior ACL injury and reconstruction, including reduced lower limb strength, poor knee joint health and reduced quality of life [9]. Skeletal muscle hypertrophy is defined as an increase in muscle mass, or the cross-sectional area (CSA) of a muscle or muscle fibre in response to physical activity [10]. Conversely, skeletal muscle atrophy is characterised by decreased muscle mass and fibre CSA [11]. Arthrogenic muscle inhibition (AMI) is a well-known complication following ACL injury and reconstruction. AMI can be grossly defined as a failure of the nervous system to activate the quadriceps muscles in the previously injured limb. Consequently, this blunting of muscle activation may lead to a lesser stimulus received by the contractile tissue during rehabilitation exercises aimed at restoring quadriceps muscle mass and function. However, the molecular mechanisms responsible for attenuated hypertrophic adaptations within the quadriceps muscles following ACL injury and reconstruction are not well understood [12–16].

Related research has largely focussed on the molecular mechanisms underlying disuse muscle atrophy following periods of immobilisation, unloading or bed rest as a result of other injuries or illnesses [17–19]. Nutritional interventions have often been utilised to effectively minimise atrophy and/or accelerate restoration of muscle mass in conjunction with other interventions following atrophy [20–22]. However, despite the known burden and consequences of ACL injuries and the impacts of poor quadriceps mass and strength on long-term health, there is currently little known about how nutritional interventions may be utilised to both attenuate loss of muscle mass and support recovery of quadriceps mass and strength throughout ACL rehabilitation and beyond. Specifically, there may be a role for nutrition in attenuating the loss of muscle early after ACL reconstruction to attenuate disuse atrophy, and optimising gains in muscle mass during early physical therapy and later phases of rehabilitation.

A better understanding of how nutritional interventions could enhance restoration of muscle mass and strength following ACL injury and reconstruction could be critically important to help reduce long-term burden and optimise patient outcomes. To effectively achieve this, the links between the potential mechanisms of quadriceps atrophy following ACL injury and reconstruction, as well as the role that nutrition can play to augment the restoration of the quadriceps muscles, needs to be expounded. Therefore, the aim of this review is to discuss nutritional interventions that may benefit patients undergoing ACL rehabilitation. To achieve this, we have reviewed the molecular mechanisms involved in muscle hypertrophy and muscle disuse atrophy in conditions that lead to under-utilisation of skeletal muscle and identified nutritional supplements with the potential to leverage these mechanisms to promote muscle hypertrophy following ACL injury and reconstruction.

## Search Strategy

To source the available literature, we searched Medline, Embase and CINAHL databases for literature assessing the effect of nutritional supplementation together with resistance training from the earliest available dates up until 2023. We also performed a manual search of references within search results. Keywords and major subject headings used in combination included ‘disused muscle atrophy’, ‘skeletal muscle disuse atrophy’, ‘muscle disuse’, ‘disuse atrophy’, ‘muscle disuse atrophy’, ‘immobilisation’, ‘dietary supplements’, ‘diet therapy’, ‘nutrition therapy’, ‘dietary supplement’, ‘supplements’, ‘nutrition’, ‘randomised controlled trial’, ‘controlled trial’ and ‘clinical trial’. It should be acknowledged that the current review did not employ a systematic search strategy, and as such, some relevant literature may not be included in this review.

## Muscle Atrophy and Rehabilitation after ACL Injury and Reconstruction

The ACL is a key stabilising ligament within the knee joint. Injury to the ACL could result in knee instability which may lead to further damage within the knee, such as cartilage injury and subsequent osteoarthritis [5, 23]. Poor outcomes related to knee function [9, 24] and risk of re-injury [25] have also been reported, even after rehabilitation following ACL injury and reconstruction. A major factor associated with the above-mentioned poor outcomes is the persistence of altered quadriceps strength and function among individuals with ACL injuries [9]. As such, a major focus of ACL injury management includes optimising recovery of the quadriceps muscles [26]. Management of an ACL injury can be divided into surgical and non-surgical approaches [27, 28]. The choice between these paths depends on various factors, including the patient’s age, activity level and extent of knee instability. Operative treatment typically involves ACL reconstruction surgery, where a graft is used to reconstruct the damaged ligament. These grafts are commonly taken from either the knee flexors (hamstring tendon) or knee extensors (patellar or quadriceps tendon) [29]. Regardless of whether a non-operative or operative approach is taken, an extensive rehabilitation program is typically needed [30]. Early management of an ACL injury is primarily aimed at reduction of pain and swelling, range of motion exercises, activation of the quadriceps muscles and proprioceptive training [31]. Rehabilitation programs following ACL reconstruction typically comprise four phases (discussed in more detail in Sect. 6), with progression through each phase dependent on clinical milestones, with ‘return to play’ activities being a late-stage goal from approximately 9 months post-surgery [32, 33]. Despite this, alterations in quadriceps strength and function is still common even after rehabilitation [7, 8].

The persistence of muscle strength deficits in individuals with ACL injuries appears to be related to a combination of different factors. Pain may directly inhibit muscle activation through nociceptive pathways, while swelling can increase intra-articular pressure which could lead to a disrupted joint receptor function [34]. Disuse atrophy from the initial injury has been reported previously [35]. The choice of ACL surgery, on its own, may also stimulate a cascade of inflammatory and metabolic responses that shift the human body into a more catabolic state [36]. This can, in some cases, last for as long as 2–4 weeks after surgery [37]. Preoperative fasting, while outside the scope of this review, may also contribute to muscle atrophy following ACL injury and reconstruction [38, 39]. The onset of AMI may also contribute to the atrophy and alteration in quadriceps function following ACL injuries [35]. Likewise, AMI could result in disruption to mechanoreceptors [40] or reduction in the excitability of alpha motor neurons, particularly those innervating the quadriceps muscles [30]. These mechanisms are thought to protect the joint from further damage but inadvertently result in reduced muscle activation and motor output from the muscles.

All of the aforementioned factors can make it challenging to address quadriceps atrophy and subsequent alterations in function following ACL reconstruction. Given the association between poor quadriceps function and poor outcomes (i.e. re-injury and early onset knee osteoarthritis), optimising quadriceps recovery is paramount. Therefore, exploring additional interventions that could enhance the restoration of the quadriceps muscles is warranted.

## Molecular Pathways and Mechanisms Underlying Skeletal Muscle Atrophy and Hypertrophy

### Disuse Atrophy

**Fig. 1:**
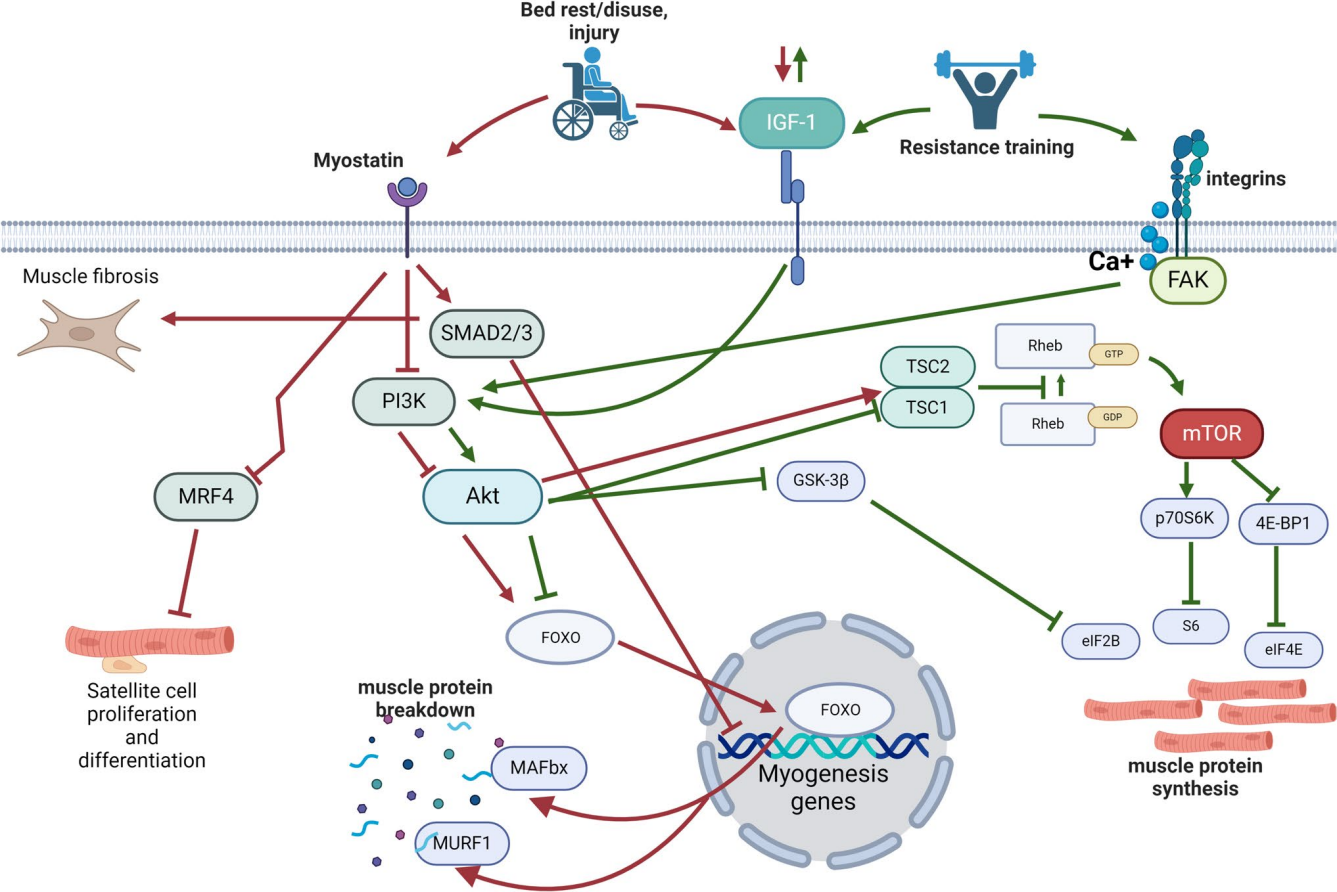
Key signalling pathways involved in muscle atrophy and hypertrophy. Resistance exercise training and muscle disuse (e.g. following injury and/or bed rest) stimulate muscle hypertrophy (green arrows) and atrophy (red arrows), respectively, and are dependent on positive and negative regulation of a number of key signalling pathways. Resistance training can increase skeletal muscle hypertrophy by activating the Akt and mTOR signalling pathways via both IGF-1 signalling and mechanical stimulation of cell membrane integrins. Activated mTOR increases muscle protein synthesis by phosphorylating a range of key protein substrates, such as GSK-3β, p70S6K and 4E-BP1. In contrast, muscle disuse results in atrophy by increasing myostatin expression, that in turn, regulates downstream myostatin signalling pathways. Myostatin negatively regulates muscle protein synthesis by inhibiting the Akt pathway, which in turn negatively regulates the mTOR signalling pathway via TSC1/2 and Rheb. Increased myostatin also leads to FOXO translocation to the cell nucleus, increasing transcription of genes involved in muscle protein breakdown. Simultaneously, myostatin signalling inhibits satellite cell proliferation and differentiation by inhibiting MRF4 and activating the Smad2/Smad3 complex, switching on genes involved in muscle wasting and leading to muscle fibrosis. Akt, protein kinase B; 4E-BP1, eukaryotic initiation factor 4E-binding protein 1; GDP, guanosine-5′-diphosphate; GTP, guanosine-5′-triphosphate; FAK, focal adhesion kinase; FOXO, forkhead box protein-O; MAFbx, muscle atrophy F-box gene; GSK-3β, glycogen synthase kinase-3 beta; mTOR, mammalian target of rapamycin; MRF4, myogenic regulatory factor-4; MuRF-1, muscle RING-finger protein-1; p70S6K, ribosomal protein S6 kinase beta-1; PI3K, phosphoinositide 3-kinase; Rheb, Ras homolog enriched in brain; Smad, receptor-regulated Smad; TSC, tuberous sclerosis proteins

Muscle atrophy occurs rapidly following periods of immobilisation, unloading or bed rest [17, 18]. Atrophy can occur as quickly as 0.5% per day [41], which equates to muscle tissue losses between 150 and 400 g from a single leg within the first 2 weeks of disuse, in the absence of contractile stimuli [42]. This rapid onset of muscle disuse atrophy is characterised by a decline in post-absorptive and post-prandial protein synthesis [43, 44]. Several signalling pathways and molecular mechanisms are known to mediate muscle growth in response to resistance exercise training and/or contribute to the development of muscle atrophy following periods of bed rest, disuse and injury (Fig. 1). Mechanistic target of rapamycin (mTOR) is a well-established central mediator of skeletal muscle protein synthesis that becomes activated in response to bouts of resistance exercise and/or protein supplementation, resulting in muscle hypertrophy [45–50]. Conversely, reduced mTOR activity, for example, in response to immobilisation and unloading following injury, results in decreased levels of protein synthesis and an imbalance in muscle protein turnover, favouring muscle protein breakdown [51–53] (Fig. 1).

Myostatin inhibits pathways involved in muscle growth upstream of mTOR, and thereby acts as a negative regulator of muscle mass [54–58] (Fig. 1). Myostatin is uniquely expressed in skeletal muscle, where its encoded protein is secreted into the plasma and binds to muscle cell receptors, triggering a series of downstream signalling pathways responsible for inhibiting muscle growth [59]. Elevated levels of myostatin have been shown to inhibit phosphorylation and activation of protein kinase B (Akt), two major consequences of which include promoting muscle protein breakdown via E3 ubiquitin ligases [60–62] and inhibiting mTOR activity [63].

Besides mTOR, activation of the ubiquitin pathway by myostatin is another mediator of muscle protein breakdown. Increased myostatin signalling within skeletal muscle results in the translocation of forkhead box-O (FOXO) transcription factors to the nucleus, promoting the expression of genes involved in muscle protein breakdown, including expression of E3 ubiquitin ligases, such as muscle RING-finger protein-1 (MuRF1) and atrogin-1/Muscle Atrophy F-box gene (MAFbx) [64–66]. This increase in the expression of specific E3 ubiquitin ligases such as MuRF1 and MAFbx leads to the ubiquitination of muscle proteins, thereby targeting their cellular degradation and contributing to muscle protein breakdown [67] (Fig. 1).

Myostatin negatively regulates the proliferation and differentiation of skeletal muscle satellite cells via myogenic regulatory factor-4 (MRF4) [68, 69] as well as fibroblast proliferation [70]. Satellite cells are muscle stem cells that remain quiescent in skeletal muscle, but become activated following muscle injury, or in response to exercise, to promote muscle growth and repair [71]. This overall reduction in the satellite cell population promotes an environment that negatively influences muscle quality by promoting fibrotic tissue formation and fat deposition [72], as well as triggering an increase in systemic inflammatory cytokines [73]. Circulating levels of inflammatory markers, such as transforming growth factor-β (TGF-β), a cytokine that can directly induce muscle atrophy [74], have been shown to be significantly increased following ACL reconstruction [73]. There are similarities between these findings and other traumatic joint injuries, whereby joint inflammation results in greater inflammation of the surrounding musculature [75, 76] Furthermore, declines in muscle protein synthesis, and potentially muscle atrophy, can result from increased inflammation in surrounding muscle tissue as a result of traumatic injury and surgery [77]. While literature on this topic is currently limited, inflammation as a result of the trauma of injury or surgery (e.g. ACL injury and reconstruction) warrants further investigation, as it cannot be captured in experimental models of disuse atrophy.

While earlier studies indicated that static markers of protein breakdown increase shortly after the onset of muscle disuse, more recent research using stable isotope techniques has demonstrated that atrophy associated with disuse is driven by declines in muscle protein synthesis, not increases in muscle protein breakdown [78, 79]. Further to this, disuse has been shown to decrease the post-prandial protein synthetic response to protein intake [42, 43, 79, 80]. Protein ingestion also increases muscle protein synthesis and skeletal muscle hypertrophy via mTOR signalling [80, 81] through downstream phosphorylation of eukaryotic initiation factor 4E-binding protein 1 (4E-BP1) and p70S6K (Fig. 1) [82]. Anabolic resistance to protein ingestion during periods of muscle disuse is a significant challenge that impairs muscle recovery and growth. Several factors contribute to this, including: (1) the altered signalling pathways mentioned above [42, 79]; (2) decreased insulin sensitivity [83], the onset of which has been linked to disuse atrophy by several studies [84, 85], within as little as 24 h of disuse [86]; and (3) changes to the expression of key amino acid transporters in skeletal muscle following disuse in an mTOR-dependent manner [87].

### Muscle Hypertrophy

Muscle hypertrophy occurs when the rate of muscle protein synthesis is greater than the rate of muscle protein breakdown [88]. However, to achieve significant muscle hypertrophy, net protein synthesis must exceed net muscle protein breakdown for weeks, or even months [89]. The main signalling pathway responsible for mediating the increase in muscle protein synthesis in response to resistance training is the PI3K–Akt–mTOR pathway [90, 91] (Fig. 1). Akt phosphorylation promotes protein synthesis, glucose uptake and lipid metabolism through regulation of its protein substrate targets such as mTOR and glycogen synthase kinase 3 (GSK-3) [92]. Evidence of increased protein synthesis can be seen within 4 h [93, 94] following a resistance exercise training session and can persist for up to 48 h following completion of exercise [95, 96].

Mechanical signals generated by muscle contraction during resistance exercise are transmitted by the extracellular matrix (ECM) via transmembrane proteins (integrins) that act as a connection between the ECM and the actin cytoskeleton within the muscle cell [97]. Mechanical stretch applied to integrins is thought to potentially activate hypertrophic signalling pathways through focal adhesion kinase (FAK) (Fig. 1) [98]. The exact mechanism by which FAK aids in regulation of muscle mass is unknown; however, it is suggested that FAK may be involved in activation of the PI3K–Akt signalling pathway and inhibition of tuberous sclerosis complex 2 (TSC2), ultimately resulting in activation of mTOR and subsequent activation of other downstream regulators of muscle protein synthesis [99]. The TSC complex acts as a GTPase-activating protein (GAP) for the Ras homolog enriched in brain (Rheb) protein, which is required for mammalian target of rapamycin complex 1 (mTORC1) activation. In the guanosine-5′-triphosphate (GTP)-bound state, Rheb binds to and activates mTORC1, which signals downstream substrate phosphorylation (Fig. 1) [100]. Additionally, activated Akt inactivates transcription factor FOXO [101]. Akt activation, particularly through the inhibition of the FOXO transcription factors, can suppress proteolytic pathways such as the ubiquitin–proteasome system and autophagy, which are responsible for protein breakdown. This suppression helps to balance the initial increase in protein breakdown, ensuring that muscle protein synthesis dominates over time, contributing to net muscle growth. However, resistance exercise can lead to an acute increase in protein breakdown, as reported by Damas et al. [89]. This increase is part of the muscle’s initial post-exercise adaptive response to facilitate muscle repair and growth. Simultaneously, the activation of the Akt pathway following resistance exercise primarily promotes muscle protein synthesis through stimulating mTOR signalling. Therefore, activation of Akt by resistance exercise induces muscle hypertrophy both by upregulating protein synthesis and suppressing protein breakdown machinery. Likewise, Akt acts as a crucial mediator of the cellular responses triggered by insulin-like growth factor 1 (IGF-1) [102, 103], which is of particular importance during periods of growth and development, such as skeletal muscle hypertrophy.

Downstream substrates phosphorylated by mTORC1 (referred to subsequently in this narrative review as ‘mTOR’ or ‘mTOR pathway’) that stimulate muscle hypertrophy include ribosomal protein S6 kinase (p70S6K), eukaryotic translation initiation factor 4E (eIF4E) and eukaryotic initiation factor 4E-binding protein 1 (4E-BP1) (Fig. 1) [104–106]. Early work by Baar et al. [47] demonstrated a strong association between p70S6K and muscle hypertrophy following 6 weeks of high-frequency electrical stimulation. More recent work has demonstrated p70S6K activation in response to skeletal muscle mechano-transduction underlying the regulation of muscle hypertrophy [107, 108]. Studies have also demonstrated that the time between successive bouts of training and muscle protein synthesis responses is modulated by eukaryotic translation initiation factor 4E-binding protein 1 (4E-BP1) [109], and that insufficient recovery between bouts of resistance exercise may impede hypertrophic gains by decreasing muscle protein synthesis via 4E-BP1.

### Impact of ACL Injury and Reconstruction on Mechanisms Involved in Muscle Hypertrophy

The precise molecular mechanisms responsible for persistent muscle atrophy following ACL injury and reconstruction are yet to be fully elucidated. Rehabilitative exercises alone are often insufficient for increasing quadriceps muscle hypertrophy following ACL injury and reconstruction [30, 110–112]. Studies that have investigated these underlying mechanisms point to the involvement of regulators upstream and downstream of mTOR [112]. Furthermore, following ACL injury and reconstruction, circulating myostatin (a key regulator of muscle protein breakdown and synthesis) levels have been found to be 50% greater compared with uninjured patients [113]. This increase in myostatin results in downstream activation of E3 ubiquitin ligases such as MuRF1 and atrogin-1/MAFbx, which have also been shown to be elevated in muscle following ACL injury and reconstruction [73, 113–115].

Moreover, myostatin has also been shown in animal models to impair muscle regeneration by negatively impacting satellite cells [116]. Satellite cell abundance likewise plays a critical role in muscles’ remodelling capacity following injury and has been shown to be decreased following ACL injury and reconstruction [117]. There is also a positive correlation between increased myostatin and increased fibro-adipogenic progenitors (FAPs) following ACL injury and reconstruction [115, 118]. These increased levels of FAPs following ACL injury and reconstruction may contribute to aberrant ECM deposition, muscle fibrosis and collagen remodelling [115].

Despite the growing incidence of ACL injury, very few studies to date have explored the molecular mechanisms underpinning quadriceps muscle atrophy following ACL injury and reconstruction. Literature to date suggests, for example, that myostatin may play a role in increasing muscle protein breakdown [73, 119]. Future studies should expand analyses to investigate additional molecular pathways and targets known to be involved in muscle protein synthesis and breakdown that may contribute to deficits in muscle function and atrophy following ACL injury and reconstruction. These future investigations will help establish whether there is a key therapeutic window for targeting this type of muscle atrophy following ACL injury and reconstruction.

## Established and Emerging Nutritional Supplements to Enhance Muscle Hypertrophy or Decrease Muscle Atrophy following ACL Injury and Reconstruction

Numerous studies have documented the role of nutritional support, including nutritional supplements, in maintaining and/or increasing muscle mass [120–122]. This evidence is primarily related to facilitating muscle hypertrophy in response to resistance training; however, building on this may be an effective strategy for increasing muscle hypertrophy and decreasing muscle atrophy following musculoskeletal injuries, and in particular, the challenges that arise following ACL injury and reconstruction [123]. Furthermore, there appears to be a crucial role for mTOR in integrating signals from a variety of inputs to regulate muscle protein synthesis and skeletal muscle hypertrophy [124].

**Fig. 2:**
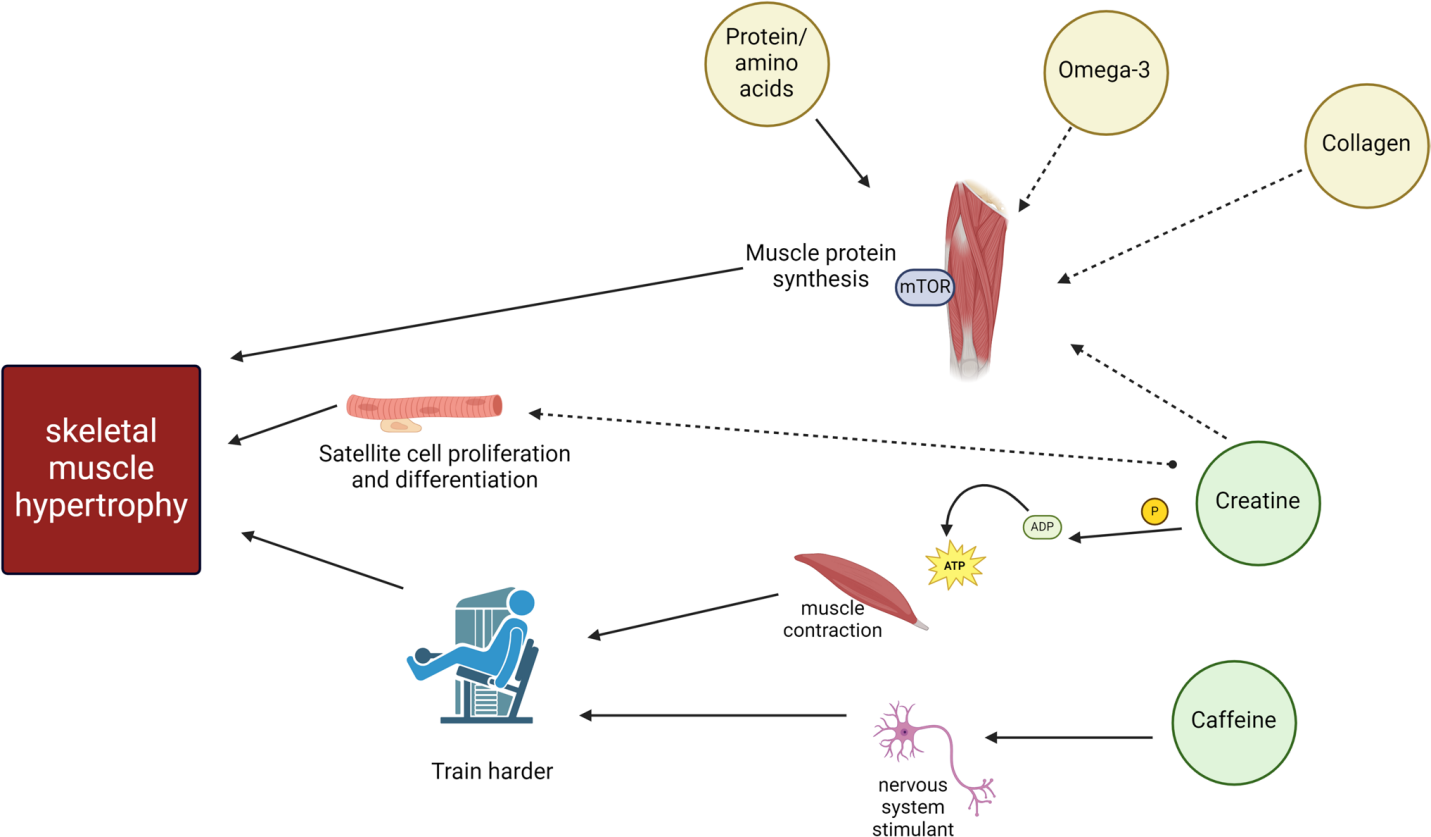
Effects of nutritional supplementation on mechanisms underlying muscle hypertrophy. There are three major mechanisms by which nutritional supplements may augment muscle hypertrophy: (1) increasing muscle protein synthesis, (2) increasing the individual’s ability to work harder and (3) increasing satellite cell proliferation and differentiation. Dotted lines indicate mechanisms in which the evidence is less established to date. ADP, adenosine diphosphate; ATP, adenosine triphosphate; mTOR, mammalian target of rapamycin; P, phosphate

There are three main mechanisms by which nutritional supplementation may support muscle hypertrophy: (1) by exerting a direct effect on muscle protein synthesis; (2) indirectly, by increasing the individual’s ability to work harder at an appropriate phase of rehabilitation when the repaired ligament is sufficiently robust. For example, the primary pathway by which by which creatine may increase muscle hypertrophy is by increasing intramuscular phosphocreatine, which donates a phosphate to adenosine diphosphate (ADP), enabling adenosine triphosphate (ATP) to replenish faster. This increased pool of intramuscular phosphocreatine increases the capacity for high-intensity exercise within the muscle, a strategy that may be of benefit in the latter stages of rehabilitation, which have a greater focus on strength and power following the returned capacity of the injured ligament to tolerate load (Table 2). However, there is evidence suggesting that creatine may also stimulate mTOR and satellite cell proliferation and differentiation [125, 126]. Similarly, caffeine is a central nervous system stimulant and may decrease perception of effort so an individual can train harder at the appropriate phase (e.g. later phase) of rehabilitation. (3) Finally, by supplementation possibly having a direct effect on satellite cell proliferation and differentiation. Figure 2 summarises the most popular nutritional supplements to date with known efficacy for augmenting muscle hypertrophy when consumed alone, or in conjunction with resistance exercise training (Fig. 2).

As discussed above, resistance training stimulates muscle hypertrophy in healthy skeletal muscle. However, following ACL injury and reconstruction, inhibition of these hypertrophic pathways occurs that either promotes skeletal muscle atrophy or hinders the normal hypertrophic responses that persists beyond rehabilitation. We propose that nutritional support may enhance rehabilitative exercise training and play a restorative regulatory role on the molecular machinery within the muscle. Before undertaking a more detailed analysis of the current evidence for each dietary or supplementation strategy, Table 1 summarises key issues and mechanisms involved in muscle hypertrophy, both in healthy skeletal muscle and during injury rehabilitation, with regard to nutritional support.

**Table 1:** Nutritional supplementation studies in experimental models of disuse atrophy and knee injury, and their impact on skeletal muscle hypertrophy and atrophy^#^

Supplement: protein and essential amino acids					
Study	Population	Atrophy model	Duration of study	Supplement	Main outcomes
Dirks et al. (2014) [86]	Healthy older volunteers (23 M) Age: 68–70 years	Single limb immobilization	5 days single leg cast	High whey protein leucine-enriched supplement (21 g protein) versus placebo Twice daily	No difference in muscle strength, size or fibre characteristics between supplement groups Muscle RING-finger protein-1 expression was higher in the protein group than placebo group
Dreyer et al. (2013) [160]	28 older adults (19 F, 9 M) Age: 68–70 years	Total knee arthroplasty	1 week prior to surgery and 2 weeks post-surgery	20 g essential amino acids twice daily versus placebo	Less quadriceps and hamstring atrophy in both legs in experimental group
Dreyer et al. (2018) [161]	39 older adults (25 F, 14 M) Age: 53–76 years	Total knee arthroplasty	1 week prior to surgery and 6 weeks post-surgery	20 g essential amino acids twice daily versus placebo	Significantly less decrease in hamstring and quadriceps muscle volume in both legs in experimental group
Edwards et al. (2020) [162]	Healthy volunteers (16 M) Age: 22–24 years	Single limb immobilization	7 days unilateral lower limb immobilization	15 g leucine/day versus 15 g placebo/day	No difference in FFM loss or leg limb muscle fibre cross-sectional area in immobilised leg between groups Within-group leg strength decreased but no difference between supplement groups No significant differences between supplement groups in myofibrillar or mitochondrial protein synthesis No significant differences between supplement groups in markers of intramuscular signalling
Holloway et al. (2019) [163]	Healthy volunteers (20 M) Age: 20–45 years	Single limb immobilisation	7 days single leg immobilisation	Novel amino acid composition (AXA2678) 23.7 g, three times daily	Supplementation attenuated atrophy and accumulation of fat during short-term disuse
^a^Laboute et al. (2013) [164]	45 athletes (32 M, 13 F) Age: 18–45 years	ACL reconstruction	2–3 weeks rehabilitation and supplementation post-ACL reconstruction	330 mg leucine capsules (one tablet in the morning and at noon, and two in the evening)	Leucine supplementation combined with resistance training increased thigh muscle diameter
Mitchell et al. (2018) [165]	Healthy volunteers (30 M) Age: 45–60 years	Unilateral lower limb immobilisation	7 weeks total 2 weeks immobilisation followed by 14 days of ambulatory recovery and 14 days of resistance training	20 g of milk protein concentrate, once daily	Muscle protein synthesis was increased only with protein supplementation Protein supplementation did not attenuate loss of muscle size and function with disuse but enhanced myofibrillar muscle protein synthesis during ambulant recovery
Ueyama et al. (2023) [166]	52 patients with primary knee osteoarthritis	Total knee arthroplasty	1 week prior to surgery and 2 weeks post-surgery Periodic follow-up for up to 2 years post-surgery	26 patients received 9 g/ day essential amino acids 26 patients received placebo	At 2 years post-surgery there were no significant differences between groups in quadriceps strength or size of rectus femoris

Supplement: creatine					
Study	Population	Study design	Duration of study	Supplement	Main outcomes
Backx et al. (2017) [167]	Healthy young men Age: 23 ± 1 years	Single leg immobilisation	7 days immobilisation	Creatine 20 g/day	Creatine supplementation prior to and during leg immobilisation had no effect on loss of muscle mass or strength
Eijnde et al. (2001) [168]	22 healthy volunteers (19 M, 3 F)	Single leg immobilisation	2 weeks immobilisation followed by 6 weeks of rehabilitation	Creatine 20 g daily during immobilisation, and 15 and 5 g daily during the first 3 and the last 7 weeks of rehabilitation	Creatine supplementation offset decline in muscle GLUT4 protein during immobilisation, and increased GLUT4 protein content during rehabilitation
Eijnde et al. (2005) [169]	22 healthy volunteers (19 M, 3 F)	Single leg immobilisation	2 weeks immobilisation followed by 6 weeks of rehabilitation	Creatine from 15 g down to 2.5 g daily	Creatine enhanced insulin-stimulated muscle glucose uptake
Hespel et al. (2001) [170]	22 healthy volunteers (19 M, 3 F)	Right leg immobilisation	2 weeks immobilisation followed by 10 weeks of rehabilitation	Creatine from 20 g down to 5 g daily	Muscle cross-sectional area and maximal knee-extension power recovered faster in creatine than placebo
Johnston et al. (2009) [171]	7 healthy males Age: 22 years	Single upper limb immobilisation	29 days	Creatine 4 × 5 g per day	Creatine supplementation maintained lean tissue mass and strength better than placebo
^a^Tyler et al. (2004) [172]	60 volunteers (33 M, 27 F) Age: 30.4 ± 1.0 years	Patients scheduled for ACL surgery	Up to 24 weeks post reconstruction	Creatine 20 g/day for the first 7 days, reduced to 5 g/day	No effect of supplementation on strength or power loss

Supplement: Omega 3					
Study	Population	Study design	Duration of study	Supplement	Main outcomes
McGlory et al. (2019) [173]	20 healthy female volunteers Age: 22 ± 3 years	Unilateral limb immobilisation	2 weeks immobilisation	4 weeks prior to immobilisation, participants consumed either 5 g/day of *n*–3 fatty acid or placebo	Decline in muscle size and mass greater in placebo than omega–3 group MyoPS was higher in the omega–3 group compared with the control group at all times

Supplement: mixed supplements					
Study	Population	Study design	Duration of study	Supplement	Main outcomes
Derave et al. (2003) [174]	33 young healthy volunteers (26 M, 7 F) Age: 18–30 years	Right leg immobilisation	2 weeks immobilisation, 6 weeks of rehabilitation	3 doses of 5 g creatine per day during immobilisation and one dose of 2.5 g creatine, 40 g protein and 6.7 g amino acids during rehabilitation	Protein did not further enhance the effects of creatine during rehabilitation
^a^Holm et al. (2006) [175]	26 ACL-injured patients (16 M, 10 F) Age: approximately 25 years	Approximately 18 months post-ACL reconstruction	12-week strength training program	10 g protein, 7 g carbohydrate compared with carbohydrate alone (17 g)	Protein and carbohydrate combined following exercise resulted in greater muscle strength than carbohydrate alone or placebo
^a^Lopez-Vidriero et al. (2019) [176]	72 ACL-injured patients (60 M, 12 F) Age: 18–55 years	Up to 90 days post-ACL reconstruction	90 days rehabilitation program	2500 mg of hydrolysed collagen, 300 mg of porcine plasma proteins with hyaluronic acid and 40 mg of vitamin C	The supplement group had a higher International Knee Documentation Committee score at 60 days follow-up compared with placebo
^a^Shaw et al. (2019) [177]	2 professional rugby athletes	Left ACL rupture	34-week rehabilitation program	10 g of gelatine with 250 mg vitamin C	Leg strength returned to a maximum at 24 and 15 weeks, respectively, with knee function returning to baseline by 30 weeks

Supplement: other					
Study	Population	Study design	Duration of study	Supplement	Main outcomes
^a^Eraslan et al. (2015) [178]	34 volunteer male athletes	Post-ACL reconstruction	8 weeks	Glucosamine 1000 mg daily	Glucosamine supplementation did not improve the rehabilitation outcomes of athletes after ACL reconstruction
^a^Barker et al. (2009) [179]	20 male volunteers	New ACL injury and reconstruction	2 weeks prior to surgery through to 3 months post-surgery	Twice-daily 200 IU vitamin E (50% D-α-tocopherol and 50% D-α-tocopheryl acetate) and 500 mg vitamin C	Supplementation did not affect limb force production up to 3 months post-surgery Supplementation increased the infiltration of inflammatory cells

M, male; F, female; FFM, fat-free mass

^#^The current review did not employ a systematic search strategy and as such, some relevant literature may not be included in this table

^a^Studies in ACL populations

### Energy Availability

**Fig. 3:**
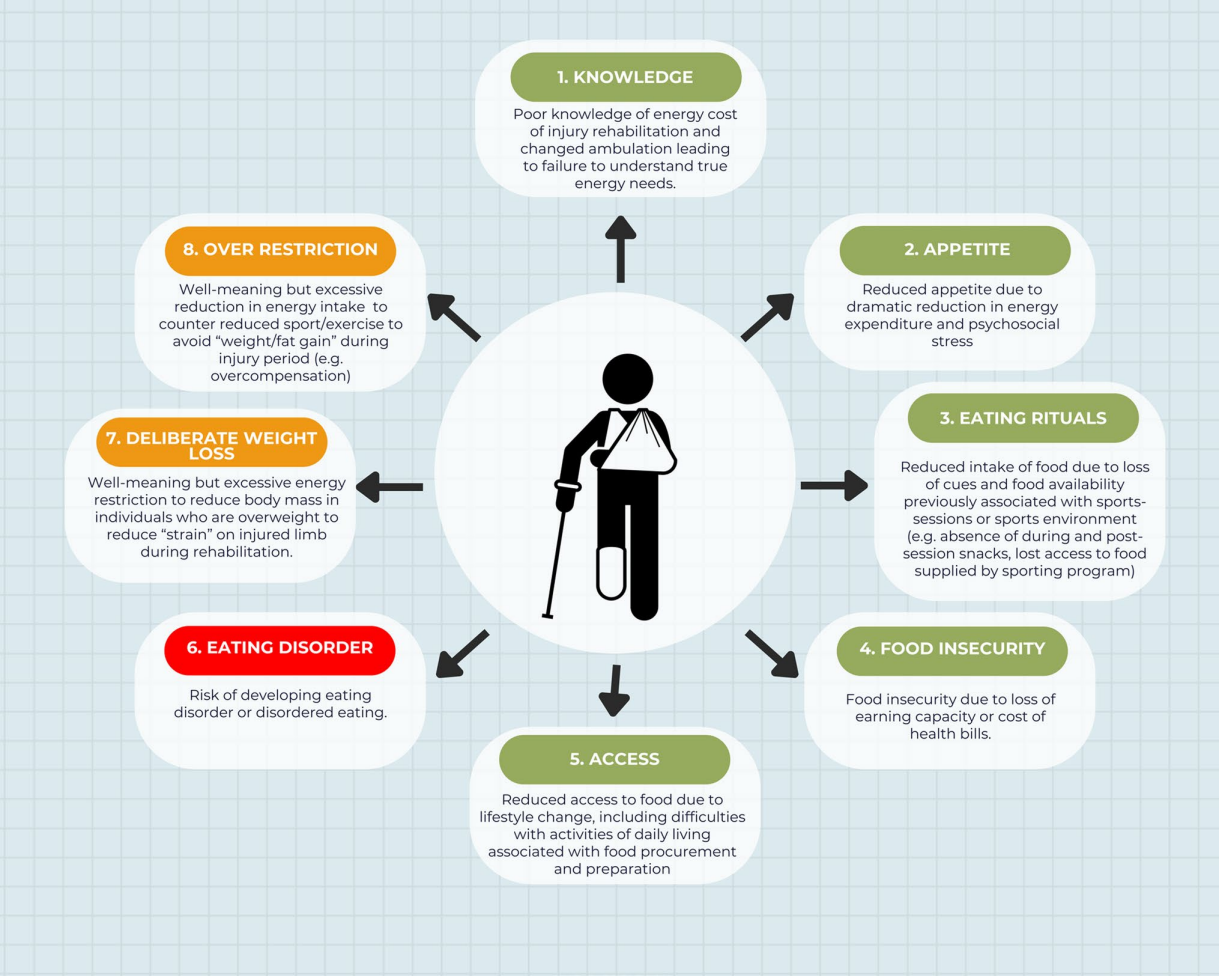
Risk factors for ‘low energy availability’ in injured populations (adapted from Burke et al. 2021). Pathological risk factors (red), intentional but misguided risk factors (orange) and inadvertent risk factors (green)

Energy availability is defined as the energy that remains to support body functions involved in health and wellbeing once the energy cost of daily exercise is removed from dietary energy intake. Mathematically, it is derived from energy intake minus exercise energy expenditure and expressed relative to fat-free mass to represent the body’s most metabolically active tissues [127, 128]. Low energy availability is a mismatch of energy intake and exercise energy expenditure, and can occur via restriction of food intake, an increase in the energy cost of exercise or a mix of both factors. Such a mismatch can occur owing to a variety of reasons that can be considered pathological, intentional but misguided behaviours and circumstantial or involuntary changes to the individual’s environment or eating behaviours [129]. Some of these factors may occur predictably in an injured individual (Fig. 3). For example, the intuitive response following injury is often to reduce energy intake with the aim of avoiding excess weight gain. However, the magnitude of such a decrease in energy expenditure following an injury with limb immobilisation (e.g. ACL injury) may be less than expected, particularly given the energy expenditure required as part of the healing process [130]. Furthermore, while there may be a reduction in physical activity following injury, factors such as the use of crutches increase ambulant energy expenditure by up to three times [131]. Alternatively, the psychosocial stress associated with a catastrophic injury, such as to the ACL, has been identified as a risk factor for developing an eating disorder [132], especially in developing and talented athletes. Ironically, this may leave a legacy of inadequate energy and nutrient support for rehabilitation.

Only a few studies have assessed the relationship between energy availability and skeletal muscle hypertrophy in response to resistance training, and these focus specifically on the impact of an energy deficit [133]. Pasiakos et al. (2010) found that an acute energy deficit of approximately 20% of estimated energy requirements in young healthy volunteers promoted 1.0 kg weight loss over 10 days and resulted in a 16% reduction in muscle protein synthesis. This occurred despite maintaining a moderate dietary protein intake, with corresponding reductions in muscle signalling downstream of mTOR, including 4E-BP1 (Fig. 1) [134]. Similarly, in a group of young trained participants, reduced mTOR and p70S6K (Fig. 1) signalling was observed, concomitant with a 30% reduction in muscle protein synthesis in response to 5 days of controlled exposure to low energy availability [135]. In this study, total protein intake remained at 1.4–1.6 g/kg body mass/day while energy availability was reduced from ~ 45 kcal/kg FFM/day to ~ 30 kcal/kg FFM/day, which equated to a daily reduction in energy intake of ~ 1900 kcal (for male participants) and ~ 1300 kcal (for female participants), respectively. However, muscle protein synthesis levels were restored to those observed during energy balance with a single resistance exercise session, and further enhanced by ingestion of 15–30 g of protein post-exercise, resulting in a 30% increase in muscle protein synthesis above energy balance at rest. These acute investigations suggest that an energy deficit may impair the molecular pathways involved in stimulating protein synthesis. Thus, maintaining energy availability may be an attractive strategy for optimising injury recovery, and important for mitigating muscle atrophy following ACL injury.

### Carbohydrate Availability

The importance of muscle carbohydrate stores (glycogen) as an energy substrate during exercise was first recognised in the 1960s [136], with subsequent research and practice confirming high carbohydrate availability (matching of total body carbohydrate stores to the fuel cost of exercise) as a key determinant of the performance of higher-intensity endurance exercise [137]. Meanwhile, glycogen availability has more recently been recognised for its regulatory role in exercise signalling pathways and the inter-organ cross-talk associated with exercise. For example, aerobic exercise undertaken with low muscle glycogen stores is associated with an upregulation of the pathways associated with mitochondrial biogenesis and the capacity for fat oxidation [138]. Here, low muscle glycogen is associated with changes in cellular osmolality, the availability of proteins with glycogen-binding domains and circulating concentrations of free fatty acids and hormones [139], which then modulate the during- and post-exercise response to endurance exercise. ‘Train-low’ strategies have been deliberately integrated into the training programs of many high-performance athletes to take advantage of potentially enhanced adaptive responses to such exercise stimulus. Anecdotally, there has been interest in implementing this practice into the ‘return to training’ strategies of injured team sport and endurance athletes, with the underlying hypothesis that greater aerobic adaptation might be achieved from a lower workload. This might either fast-track the restoration of conditioning following an injury break, or protect the rehabilitating limb by a more gradual return to load. However, there is other evidence to suggest that carbohydrate restriction increases protein oxidation, thereby limiting essential amino acid availability [140]. In fact, in some studies of athletic populations, carbohydrate restriction has been shown to blunt muscle hypertrophy [140], which is counter-productive for the most important part of rehabilitation.

### Protein and Essential Amino Acids

Protein is a macronutrient composed of amino acids, essential for a range of physiological functions, including skeletal muscle growth and maintenance. The recommended dietary allowance for protein intake in adults is 0.8 g/kg/day [141]. This amount increases to 1.0–1.2 g/kg/day for older adults to help limit age-related declines in muscle mass [142, 143], and can be as high as 2.0 g/kg/day for athletes to support metabolic adaptation, repair and remodelling [144].

Increased amino acid availability can stimulate muscle protein synthesis [82], even under resting conditions, and subsequently aids in maintaining the balance between muscle protein synthesis and breakdown [145]. Amino acid intake alone directly increases muscle protein synthesis, although its effects are transient, lasting only hours [146]. When combined with resistance training, however, amino acid supplementation increases muscle protein accretion for up to 72 h [83, 146]. Interest in protein and its role in muscle health has remained strong and continues to grow, as reflected in research from the past decade, leading to the widespread use of techniques to measure acute changes in muscle protein synthesis in response to the combination of resistance exercise stimuli and dietary manipulation of blood amino acid concentrations [147]. The ‘leucine trigger’ hypothesis, coined to explain the post-prandial regulation of muscle protein synthesis, suggests that the dose of leucine ingested and subsequent blood leucine concentrations determine the magnitude of the post-prandial post-exercise muscle protein synthesis response [148, 149].

Such studies underpin recommendations about the optimal timing, type, spread and dose of protein intake [150]. More recently, advancements in the application of stable isotope methodology, and interest in their translation to longer-term outcomes (e.g. gains in muscle mass and strength), have suggested that if total protein intake is > 1.6 g/kg/day [151, 152], variables such as protein timing and dose may not exert major effects. However, the impact of protein timing and frequency on muscle hypertrophy may be more significant when protein or total energy availability is restricted [135], or in special populations or scenarios with protein/anabolic resistance. Both scenarios may be in effect in individuals with ACL injuries and warrant further investigation.

**Fig. 4:**
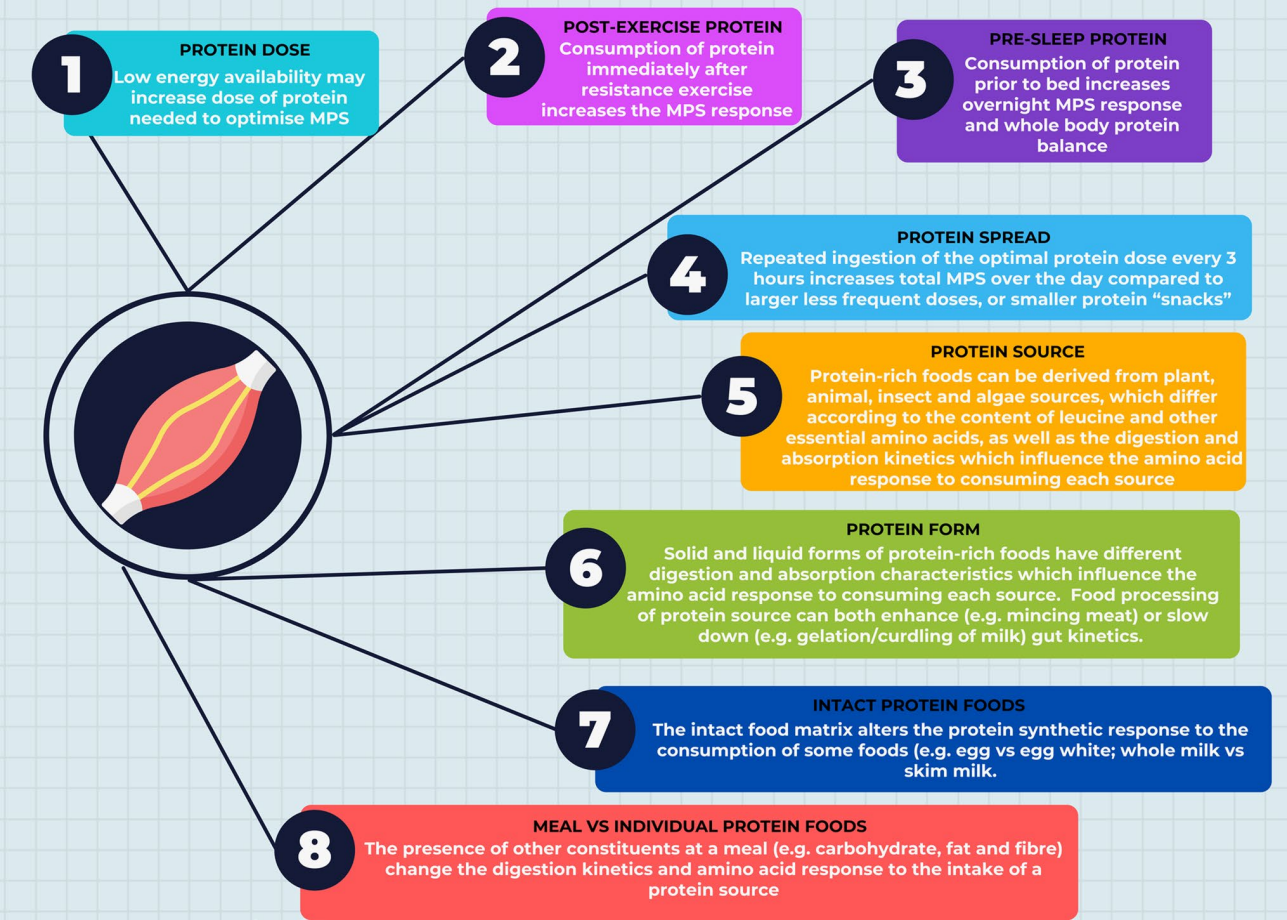
Dietary factors that may affect muscle protein synthesis (MPS) under special conditions such as reduced energy availability or anabolic resistance. Protein dose [135], post-exercise protein [239], pre-sleep protein [240], protein spread [241], protein source [242, 243], protein form [244–246], intact protein foods [247] and meal versus individual protein foods [248]

Older populations are known to experience anabolic resistance due to normal aging, requiring greater doses of protein (35–40 g) compared with younger adults (20 g) to maximally stimulate muscle protein synthesis [153, 154]. In these instances, strategies that amplify the response to protein in the face of anabolic resistance such as a high protein and leucine-enriched nutritional supplementation [155] have shown favourable results in stimulating the molecular pathways involved in muscle hypertrophy. Such strategies may also be beneficial following ACL injury and reconstruction, where a blunted post-prandial protein synthetic response to protein ingestion is seen in response to muscle disuse. Given the varied ways in which protein is utilised to augment muscle hypertrophy or overcome muscle atrophy, there remains an opportunity to maximise the overall response to protein supplementation through a variety of strategies, which may translate to improved outcomes following ACL injury and reconstruction. These may involve taking advantage of characteristics such as the amount of leucine and essential amino acids in the protein source, digestion and absorption features which alter blood amino acid profiles following the ingestion of these sources and the timing of intake over the day; such features are summarised in Fig. 4.

Although food sources of protein represent the most significant source in the diet, the use of amino acids and their derivatives, either as isolated supplements or via fortification of food products, should also be considered. Ingestion of essential amino acids (EAAs) has been shown to increase muscle protein synthesis, which is associated with increased mTOR pathway signalling [80, 81] and subsequent downstream phosphorylation of 4E-BP1 and p70S6K (Fig. 1), thus stimulating muscle hypertrophy [82]. One particular study reported increased phosphorylation of Akt, mTOR, 4E-BP1 and S6K1, and decreased phosphorylation of eEF2 (Fig. 1), in association with a significant increase in muscle protein synthesis following ingestion of leucine-enriched essential amino acids and carbohydrates [81]. Finally, leucine-fortified protein sources have been shown to improve body composition and strength as well as attenuate loss of muscle mass in older adults [156–159].

Protein and amino acids are a promising option for stimulating hypertrophy following muscle disuse, given their known capacity to stimulate muscle protein synthesis when combined with resistance training while inhibiting muscle protein breakdown. However, at the present time there is no optimally defined method or quantity that is effective at augmenting disuse atrophy, and certainly even less in models of ACL injury and reconstruction. Several studies have sought to identify a role for protein in augmenting muscle hypertrophy following disuse, although the heterogeneity of disuse/injury models, protein/amino acids ingested and participant ages, in addition to small sample sizes, makes it difficult to translate findings into functional guidelines (Table 1).

### Creatine

Creatine is a widely studied supplement, with numerous studies having shown that creatine supplementation increases muscle hypertrophy when combined with resistance training (Table 1). Phosphocreatine, or creatine, acts as a donor of phosphate to adenosine diphosphate (ADP) to produce adenosine triphosphate (ATP) required for energy production [180]. Different to protein or amino acid supplementation, the primary mechanism by which creatine supplementation increases muscle hypertrophy is improved exercise performance at high intensity, owing to increased intramuscular creatine [181, 182]. This increased creatine plays a critical role in energy metabolism, particularly in high-intensity, short-duration activities (e.g. resistance training) that rely on the rapid production of ATP [183]. By enhancing energy availability and promoting efficient cellular energy metabolism, creatine may support faster recovery between exercise bouts and attenuate the development of muscle fatigue, enabling an individual to train harder, thereby enabling greater opportunity for hypertrophy [184].

Creatine supplementation has also been reported to affect anabolic signalling, although the mechanisms underlying these effects are not fully understood and may involve multiple pathways [185]. Creatine supplementation has been shown to stimulate the Akt–mTOR signalling pathway (Fig. 1) and increase phosphorylation of the downstream substrates p70S6K and 4E-BP1 [185, 186]. Likewise, creatine may also have a stimulatory effect on satellite cell proliferation via MRFs. In a study of healthy young males who ingested creatine in conjunction with resistance exercise training over 16 weeks, there were significantly more satellite cells detected in skeletal muscle compared with participants who received protein supplementation paired with resistance training and resistance training alone [126].

Creatine supplementation has also been shown to stimulate markers of muscle hypertrophy when combined with a 6- [169, 174] or 10- [168, 170] week rehabilitative resistance training program, following disuse, to a greater extent than resistance training alone in populations of young adults. Eijnde et al. [168] administered 20 g/kg of creatine or placebo to a young adult population during 2 weeks of lower limb immobilisation, followed by incrementally smaller doses of 15 g/day (weeks 1–3) and 5 g/day (weeks 4–10) while completing a heavy resistance training program. No difference was observed during immobilisation; however, molecular changes within the muscle of the immobilised leg suggested that when combined with resistance training, creatine had a hypertrophic effect [168]. Similar studies have equally demonstrated the hypertrophic effects of creatine during the rehabilitative resistance training phase [167, 169, 170, 174]. Other studies have also demonstrated promising results in mitigating the effects of muscle disuse or immobilisation. These include a study by Johnston et al. [171] in which creatine supplementation preserved muscle mass, strength and endurance after 7 days of upper limb immobilisation in creatine-naïve men, compared with an isocaloric placebo [171]. Creatine loading is a practice frequently used by athletes and has been shown to decrease inflammatory markers [183]. While high-intensity exercise and heavy loads are typically not feasible during the early phase of rehabilitation following ACL injury and reconstruction, creatine supplementation may still be an effective strategy for preserving muscle mass during this phase.

### Omega-3 Polyunsaturated Fatty Acids

Omega-3s are a class of long chain polyunsaturated fatty acids known for their beneficial effects on human health such as enhanced cognitive function [187], reduced inflammatory mediators [188] and improved blood lipid profiles [189]. Most research on omega–3 fatty acids has utilised eicosapentaenoic acid (EPA)- and docosahexaenoic acid (DHA)-containing supplements, with current recommended daily intake, by the National Academy of Medicine and the Australian National Heart Foundation, being approximately 250–500 mg/day [190].

Omega-3s have a range of human health benefits, including reducing inflammation [187–189]. Skeletal muscle atrophy has been linked to inflammation in other disease states [191, 192], and thus represents a critical pathway to explore further. Early animal studies have also suggested a potential role for omega-3 supplementation in stimulating muscle protein synthesis and insulin signalling within skeletal muscle by activating the Akt–mTOR–S6K1 pathway (Fig. 1) [193]. Subsequent human studies examining the role of omega–3 fatty acids on muscle protein synthesis have resulted in inconsistent findings. One study found that 4 weeks of supplementation with 5 g/day in healthy young males increased mTOR signalling in skeletal muscle [194]. This was congruent with findings from another study where consumption of 2 g/day of fish oil combined with resistance training in a population of older women caused greater improvements in muscle strength than resistance training alone [195]. However, the number of studies on omega-3 fatty acids in injured populations is currently limited (Table 1) to one single limb immobilisation study demonstrating attenuation of skeletal muscle atrophy [173]. In contrast, other studies failed to demonstrate any effect of omega-3 supplementation on muscle protein synthesis [196, 197]. Da Boit et al. [197] suggest there may be age- and sex-related differences that influence the efficacy of omega-3 supplementation. Nonetheless, further research is warranted to better elucidate the potential for omega-3s to stimulate mTOR and muscle protein synthesis molecular machinery in skeletal muscle and better understand any beneficial effect on skeletal muscle during ACL injury and reconstruction rehabilitation.

### Collagen

Collagen is the most abundant protein in the human body, accounting for approximately one-third of total body protein. It is the key tensile element of ligaments and tendons, contributing to strength, rigidity and regulation of mechanical forces [198]. Collagen is also an important component of the ECM in skeletal muscle and tendons [199]. However, while rich in non-essential amino acids, such as proline and glycine, collagen is relatively lower in essential amino acids such as leucine, a known stimulator of muscle protein synthesis. Collagen has a triple-helix structure and is enzymatically hydrolysed, degrading collagen into smaller bioactive peptides in its primary supplemental form [200]. Currently, collagen supplements are sourced from bovine, porcine, marine and poultry hydrolysed collagen [201].

Collagen peptide supplementation has gained momentum in recent years and may be a potential regulator of protein metabolism in contractile fibres and phosphorylation of the PI3K–Akt and mitogen-activated protein kinase (MAPK) pathways [202]. As a relatively new supplement compared with others discussed above, collagen peptides present an opportunity for further research to better understand their role in maintaining muscle mass and strength, and indeed whether there is a role in rehabilitation following ACL injury and reconstruction. Several studies have reported improved recovery from muscle damage following collagen supplementation [203]. Collagen peptides have been shown to increase fat-free mass and leg strength in untrained men [204] and similarly in pre-menopausal women [205]. However, the impact of collagen peptides on skeletal muscle remains poorly understood, with mixed outcomes observed. Recently, collagen peptide supplementation combined with resistance training was shown to be associated with greater hypertrophy of gastrocnemius muscle when combined with a 14-week high-load resistance training program, compared with resistance training alone [206]. Centner et al. [207] found that high-load leg extension in combination with 15 g of collagen peptides upregulated gene expression related to the mTOR pathway compared with placebo. Conversely, a study comparing the effect of 30 g/day collagen peptides with the same amount of whey protein found there was a greater increase in muscle protein synthesis in the whey protein group [208]. Muscle protein synthesis was also elevated at rest and up to 4 h later, compared with the collagen group, where elevated muscle protein synthesis was only observed during resistance exercise. Likewise, Jacinto et al. [209] compared whey protein with leucine-matched collagen peptides combined with resistance training over 10 weeks in young untrained adults and observed greater increases in muscle thickness in the whey protein group. Whilst the correlation between collagen peptide supplements and muscle protein synthesis is mixed, there may be a role for collagen peptides in transmission of force to the muscle as a result of increased tendon cross-sectional area [210]. However, recent studies have shown that supplementation of male recreational athletes with 15 g hydrolysed collagen peptides during 1 week of resistance exercise training [211], as well as male and female recreational athletes with 30 g collagen protein during recovery from a single session of resistance exercise [212], does not increase muscle connective protein synthesis rate.

A further mechanism by which collagen peptides may be beneficial involves glycine, a key amino acid within collagen peptides, which constitutes one-third of collagen protein’s amino acid residues [213]. In a model of skeletal muscle inflammation, glycine supplementation was shown to be effective at restoring the anabolic response within anabolic-resistant muscle [214]. Glycine supplementation reduced signalling associated with protein breakdown, and counteracted anabolic resistance via the mTOR pathway (Fig. 1) [214]. Another animal model demonstrated an anti-inflammatory role for collagen peptides via glycine-gated chloride channels [215]. A short-term human trial of glycine alone was not able to replicate the anti-inflammatory properties of collagen supplementation compared with placebo [203]. Kitakaze et al. [216] demonstrated increased activation of the mTOR pathway, in response to hydroxyprolyl-glycine, a collagen-derived dipeptide. Therefore, it remains to be elucidated whether there is a role for collagen peptides, and what that may be, in the context of skeletal muscle hypertrophy, particularly following ACL injury and reconstruction.

### Caffeine

Caffeine is the most widely consumed supplement in the world, with an extensive body of research on its capacity to enhance the performance of a range of endurance, sustained high-intensity and team sports [217], primarily owing to its role as an adenosine receptor antagonist. There is also evidence of its capacity to support the completion of a resistance exercise session, allowing the athlete to complete more work owing to increased motivation, reduced pain and soreness and a masking of fatigue [218, 219]. The proposed mechanism underlying the effect of caffeine on muscle hypertrophy is not via a direct effect on muscle protein synthesis, but via an indirect effect of an increase in the training load during resistance (or other rehabilitation) sessions. Although the ACL rehabilitation program is primarily targeted at managing the load experienced by the repaired ligament, there is a possibility that at least some of the failure to regain pre-injury muscle size and strength is because other aspects of deconditioning reduce the athlete’s overall training load. It is possible that the use of caffeine to allow the athlete to train harder, while within the load limits of the repaired ACL, may be effective in regaining pre-injury characteristics when applied during the later stages of rehabilitation. Accordingly, this remains a promising area of opportunity for future research, particularly in injured populations such as following ACL injury and reconstruction.

### Mixed Supplementation Models

Optimising nutritional supplementation strategies and combinations for injury recovery remains an area of opportunity. As such, a number of studies have sought to combine multiple nutritional supplements. Some of these studies in populations with ACL injury or experimental models of disuse showed promising results when combining protein with carbohydrate intake [175, 220]. In particular, 26 male and female participants with prior ACL injury participating in 12 weeks of lower limb resistance training sessions achieved significantly greater quadriceps muscle hypertrophy and peak torque when ingesting a combination of essential amino acids and carbohydrate supplementation compared with carbohydrate alone or placebo [175]. Likewise, 13 healthy male participants supplemented with essential amino acids and carbohydrates during 28 days of bed rest maintained lean leg mass and demonstrated higher muscle fractional synthetic rate and attenuated loss of strength compared with the control group [220]. However, other studies of co-ingestion of carbohydrates and protein in healthy populations following resistance training have had mixed results [221–223]. These discrepancies may be partially a result of the different time courses employed between the studies in models of disuse [175, 220], which examined the effects of supplementation over a period of weeks, compared with acute intervention studies in healthy populations [222, 223].

A mixed supplement approach poses several challenges. For example, it can be difficult to differentiate any observed effects between supplements without a comparison of each supplement independently. Furthermore, there may be either synergistic or antagonistic effects between supplements. This challenge was addressed in an acute study of ten healthy male and female participants in which subjects participated in a bout of lower limb resistance exercise before consuming one of three drinks containing: amino acids, carbohydrates or a combined amino acid and carbohydrate drink, 1- and 2-h post-exercise [221]. The investigators found that the combined effect on net muscle protein synthesis of carbohydrate and amino acids following resistance exercise is approximately equivalent to the sum of the independent effects of either supplement consumed alone. This particular study therefore provides a rationale for studies to examine both combined and independent effects of supplementation where mixed supplements are being tested.

## Outcomes of Nutritional Supplementation in Populations with ACL Injury and Reconstruction

Populations with ACL injury remain a highly under-studied area of research, despite the known poor outcomes and persistent quadriceps atrophy occurring after ACL injury and reconstruction. There has been limited work to date using nutritional supplementation adjunct to physical therapy to improve outcomes in ACL injury populations.

A total of seven studies to date have specifically assessed the effect of nutritional supplementation on the quadriceps muscles following ACL injury and reconstruction (Table 1) [164, 172, 175–179]; three of these studies have shown some benefit of combining supplementation with rehabilitation [164, 175, 177], while the other four did not demonstrate any effect. It is inherently challenging to draw overall conclusions from the existing studies owing to the heterogeneity of the selected nutritional supplements and variation in sample sizes. For example, Shaw et al. (2019) demonstrated successful outcomes combining 34 weeks of rehabilitation with vitamin C and gelatine [224]. However, the total sample size comprised only two professional athletes. Meanwhile, Lopez-Vidriero et al. (2019) did not detect any benefit from the use of a combined supplement containing vitamin C with hyaluronic acid and plasma proteins in a much larger cohort of 72 participants over a similar timeframe [176].

When taken together with models of disuse in healthy participants, there appears to be an overall positive effect of supplementation during the rehabilitation period (Table 1), suggesting a possible role for amino acids, creatine and omega-3. Likewise, there are studies demonstrating a positive effect on preservation of lower limb muscle mass in older populations undergoing orthopaedic surgery [160, 225]. However, there appear to be multiple factors at play; thus whether these findings apply to cases of ACL injury and reconstruction, which is more common in younger populations [2], warrants further investigation.

## Proposed Concurrent Nutritional Support during ACL Rehabilitation

The ultimate objective of ACL rehabilitation is to restore knee strength and function, address common psychological barriers to resuming activity participation, prevent further knee injury and degeneration (e.g. knee osteoarthritis) and optimise long-term quality of life [26]. Given the importance of quadriceps strength in the abovementioned outcomes, optimising rehabilitation to address quadriceps atrophy and weakness following ACL injuries is paramount. Few studies have investigated the potential for nutritional supplementation to attenuate muscle atrophy or enhance hypertrophy following ACL injury and reconstruction specifically. Therefore, it would appear reasonable to use the existing evidence in other similar populations to guide initial recommendations, even though much more work is still needed, specifically in ACL-injury populations to validate and optimise these recommendations.

**Fig. 5:**
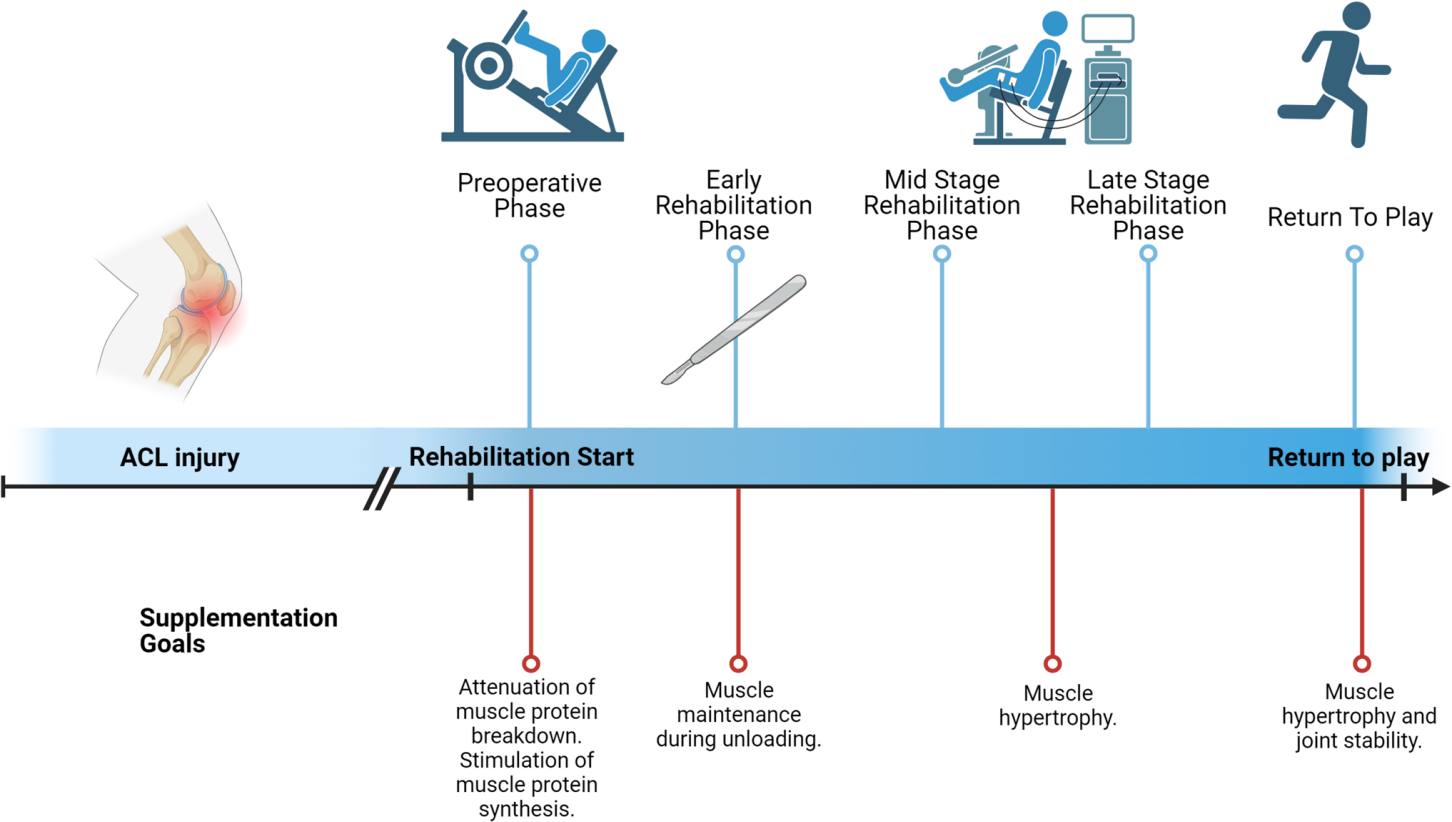
Nutritional supplementation goals complementary to the phases of rehabilitation training. The timeframe for a patient to move through each of the five phases of rehabilitation training following ACL injury is determined by their progress and reaching key milestones. As such, the nutritional requirements of the patient through each phase similarly align with the goals of each phase. Matched nutritional supplementation may enhance patient outcomes through each phase

Using current practices in the rehabilitation process, we propose potential targets and mechanisms for a nutritional strategy. Evidence-based rehabilitation programs following ACL reconstruction are typically composed of different phases [26, 32, 33, 226]. Progression through each phase is dependent on achieving specific clinical milestones (Table 2) [26]. A nutritional strategy complementary to the phases of rehabilitation could support and enhance patient outcomes and progress by providing nutrients that opportunistically target hypertrophic pathways following ACL injury and reconstruction (Fig. 5). While there is limited research translating nutritional interventions to practice following injury more broadly, and indeed ACL injury and reconstruction specifically, the available literature suggests there may be some benefit to nutritional supplementation following orthopaedic surgery. For example, one study by Dreyer et al. (2013) demonstrated that consuming 20 g of EAAs twice a day for 7 days before and 6 weeks after total knee arthroplasty attenuated quadriceps muscle atrophy compared with placebo [160].

**Table 2:** Potential nutritional supplementation to optimise rehabilitation following ACL injury and reconstruction. This strategy should be implemented in conjunction with dietary patterns which maintain adequate energy and macro/micro-nutrient availability

Phase	Timeframe	Rehabilitation goals [26, 32, 33, 226]	Supplement	Mechanism of action
Pre-operative phase	~ 2–4 weeks prior to surgery	Minimise inflammation Regain full range of active and passive motion Quadriceps strength symmetry Resistance training to improve muscle mass and overall strength	Whey protein following rehabilitative exercise	Increase muscle protein synthesis
			Omega-3 fatty acids	Decrease inflammation and increase muscle protein synthesis
			Preoperative creatine loading	Preservation of muscle mass
			Total combined dietary and supplemental protein intake Pre-operative carbohydrate supplement taken on the day of surgery	Maintaining muscle protein synthesis
Early rehabilitation phase (includes acute phase)	2 weeks to 3 months after surgery	Inflammation management (surgical site pain and swelling) Regain active and passive knee range of motion Early quadriceps activation Regain overall muscle strength Gait retraining with focus on movement quality	Omega-3 fatty acids	Decrease inflammation and increase muscle protein synthesis
			Whey protein following rehabilitative exercise Increase total protein target Collagen peptides daily prior to rehabilitative training	Maintaining muscle protein synthesis
Mid-stage rehabilitation phase	3–6 months after surgery	The knee should be free of swelling and pain during this phase Restoration of quadriceps strength symmetry Continuous recovery of overall strength Return to running Introduction of low level to high level plyometrics	Whey protein following rehabilitation exercise Increase total combined dietary and supplemental protein intake Collagen peptides prior to rehabilitative training	Increase muscle protein synthesis
			Creatine	Increase exercise capacity at maximum intensity by increasing ATP production to enable the patient to ‘train harder’ within the capacity of the rehabilitated ligament
Late-stage rehabilitation phase	6–9 months after surgery	Goals will be individualised on the basis of patient’s specific goals or demands. If return to sport is the goal, then additional focus on physical and mental readiness is added Restoration of quadriceps strength symmetry Continuous recovery of overall strength with transition to power production Plyometric training with external stimulus (e.g. perturbation) Graduated return to low level play/practice	Whey protein following rehabilitation exercise Collagen peptides prior to rehabilitative training	Increase muscle protein synthesis
			Creatine Caffeine	Increase exercise capacity at maximum intensity by increasing ATP production to enable the patient to ‘train harder’ Increase capacity to undertake rehabilitation or return to play exercise tasks, within the capacity of the tolerable load of the injured ligament, noting that loss of conditioning during inactivity may be the new limiting factor in completing the training program
Return to play/sports/performance (ongoing management)	9 + months after surgery	Involves maintenance exercises as patient returns to normal activities or full sport participation		This is beyond the scope of this review

The following proposal is preliminary in nature and is based on the current understanding of the mechanisms of muscle hypertrophy and atrophy [130, 227]. More research specific to ACL injury populations is needed to translate literature into refined clinical protocols.

The pre-operative phase of rehabilitation, often termed prehabilitation, provides a window of opportunity to optimise the physical and functional capacity of skeletal muscle prior to surgery, in which a patient is able to perform exercise. Although not yet widely practiced, prehabilitation includes medical optimisation, physical exercise, nutritional support and psychological support provided by a multidisciplinary team [228, 229]. Pre-existing nutritional status [230], including pre-operative fasting [231], can impact post-operative outcomes. Therefore, nutritional strategies employed during this phase of ACL reconstruction are two-pronged: to increase the efficacy of prehabilitation exercises in augmenting muscle hypertrophy and providing nutritional support immediately prior to surgery. In the case of the former, protein supplementation, which has been shown to increase muscle protein synthesis, particularly in combination with resistance exercises, may be of benefit during this phase. Research suggests that ∼ 20 g (or approximately 0.24–0.30 g/kg of body weight) of a high-quality protein is required to maximise muscle protein synthesis following resistance exercise [151, 232]. Wall et al. (2015) suggest that daily protein intake of 1.6–2.5 g/kg of body weight may be required to attenuate muscle atrophy during disuse [227].

During the post-operative, or acute phase (2–6 weeks post-surgery), there is a period of unloading, or reduced load. It is expected that there may be some muscle atrophy during this time [233]. Therefore, the nutritional goals during this phase should focus on strategies that are effective at promoting muscle protein synthesis and minimising muscle protein breakdown. On the basis of the current literature reviewed above, supplementation with protein, creatine and omega-3s (Table 2) appear to be the most promising avenues for further investigation.

Subsequent phases of ACL rehabilitation following surgery focus on regaining muscle strength. Following ACL injury and reconstruction, changes in expression of muscle fibre types and architecture, as well as changes in activity within molecular pathways, are seen and reviewed elsewhere by Lepley et al. [35]. These muscle fibre changes may be the result of disuse and altered activation patterns of the quadriceps muscles, owing to their reliance on continuous activation [234]. While strength training during rehabilitation is often used, and there are similarities between rehabilitation and ‘strength training’, muscle activation during resistance training and rehabilitation exercises can differ significantly. Resistance training typically involves high-intensity, progressive overload to maximise strength and hypertrophy [235]. In contrast, rehabilitation exercises typically focus on low-to-moderate intensity, emphasising neuromuscular control and joint stability with lower muscle activation levels [30]. It is not until the latter stages of rehabilitation that exercises aimed at increasing muscle hypertrophy are employed (Table 2).

Protein supplementation, for example, is an obvious prospect for increasing muscle protein synthesis during this phase [236, 237]. However, on the basis of our review of literature, supplements such as creatine [96, 238], omega-3 polyunsaturated fatty acids [238] and collagen [202, 203] have also emerged as potential candidates.

In accordance with the dosage and timing of supplementation in the literature, 20 g of a high-quality protein supplement following rehabilitative exercises may increase muscle protein synthesis. Additionally, the use of creatine in the intermediate phase (6–16 weeks post-surgery) could be considered at the dose of 5 g, four times daily, reducing to 3 g per day after the first 7 days. Whilst the goal of protein supplementation is increasing muscle protein synthesis, the addition of creatine aims to increase ATP supply within the muscle and maximise training.

Supplementation with 10 g of collagen peptides throughout rehabilitative training may also be worthwhile considering. This is to ensure that the collagen-rich ECM has a sufficient availability of substrate required during mechanical loading as a result of resistance training [177].

The final phase of ACL rehabilitation is ‘return to play’, or ongoing management. Nutritional recommendations, including supplementation for this phase, are sport/activity specific. Whilst there may be supplements that aid in maintaining joint health, they do not relate directly to muscle atrophy and thus fall outside of the scope of this review.

## Conclusions

Muscle disuse following ACL injury and reconstruction results in a loss of muscle mass and strength that is persistent and carries long-term consequences such as accelerated deterioration of knee joint cartilage [5], reduced lower limb strength and poor knee joint health [9]. Thus, it is important to find strategies that can be safely implemented to recover muscle deficits within the lower limb.

This review outlines the current literature and provides a series of practical considerations for nutritional interventions that may aid in augmenting muscle atrophy following ACL injury and encourage muscle hypertrophy before reconstruction and during the stages of rehabilitation. Appropriate nutrient availability during exercise or periods of stress has the clear potential to positively impact muscle hypertrophy. However, to clearly define the synergy between nutritional supplementation and existing rehabilitative protocols, further studies are needed in populations with ACL injury.

## Declarations

### Availability statement

All data generated or analysed during this study are included in this published article.
